# Supplementary material for: Quantifying concentration distributions in redox flow batteries with neutron radiography
Source: Nat Commun. 2024 Sep 5;15:7434. doi: 10.1038/s41467-024-50120-7 (PMC11377732; doi:10.1038/s41467-024-50120-7)
Supplement: Supplementary file 1 — Supplementary Information [file 41467_2024_50120_MOESM1_ESM.pdf]

Supporting Information
Quantifying Concentration Distributions in Redox Flow Batteries with Neutron Radiography

Rémy Richard Jacquemond[1,2,=], Maxime van der Heijden[1,=], Emre Burak Boz[1,3,=], Eric Ricardo Carreón Ruiz[4], Katharine Virginia Greco[5,6], Jeffrey Adam Kowalski[5,6], Vanesa Muñoz Perales[7], Fikile Richard Brushett[6], Kitty Nijmeijer[2,8], Pierre Boillat[4,9], Antoni Forner-Cuenca[1,3,6,*]

[1]Electrochemical Materials and Systems, Department of Chemical Engineering and Chemistry, Eindhoven University of Technology, P.O. Box 513, 5600 MB Eindhoven, The Netherlands

[2]DIFFER - Dutch Institute for Fundamental Energy Research, P.O. Box 6336, 5600 HH5612 Eindhoven, The Netherlands

[3]Eindhoven Institute for Renewable Energy Systems, Eindhoven University of Technology, P.O. Box 513, 5600 MB Eindhoven, The Netherlands

[4]Electrochemistry Laboratory, Paul Scherrer Institut, Forschungsstrasse 111, CH-5232, Villigen PSI, Switzerland

[5]Joint Center for Energy Storage Research, Massachusetts Institute of Technology, Cambridge, Massachusetts 02139, United States

[6]Department of Chemical Engineering, Massachusetts Institute of Technology, Cambridge, Massachusetts 02139, United States

[7]Department of Thermal and Fluids Engineering, Universidad Carlos III de Madrid, 28911 Leganes, Spain

[8]Membrane Materials and Processes, Department of Chemical Engineering and Chemistry, Eindhoven University of Technology, P.O. Box 513, 5600 MB Eindhoven, The Netherlands

[9]Laboratory for Neutron Scattering and Imaging, Paul Scherrer Institut, Forschungsstrasse 111, CH-5232, Villigen PSI, Switzerland

[=] These authors contributed equally to the work
*Corresponding author: a.forner.cuenca@tue.nl, +31 (0) 6 202 02 775

Supplementary Note 1 – Concentration profiles at a lower flow rate **3**
Supplementary Note 2 – Detailed electrochemical plots **4**
NEUTRA - PF_6^- experiment........ 4
NEUTRA - BF_4^- experiment 5
ICON - BF_4^- experiment 6
Supplementary Note 3 – Experimental design considerations **7**
Reactor materials and housing 7
Electrolyte composition 7
Supplementary Note 4 – Image processing **10**
NEUTRA 10
References **10**

Supplementary Note 1 – Concentration profiles at a lower flow rate

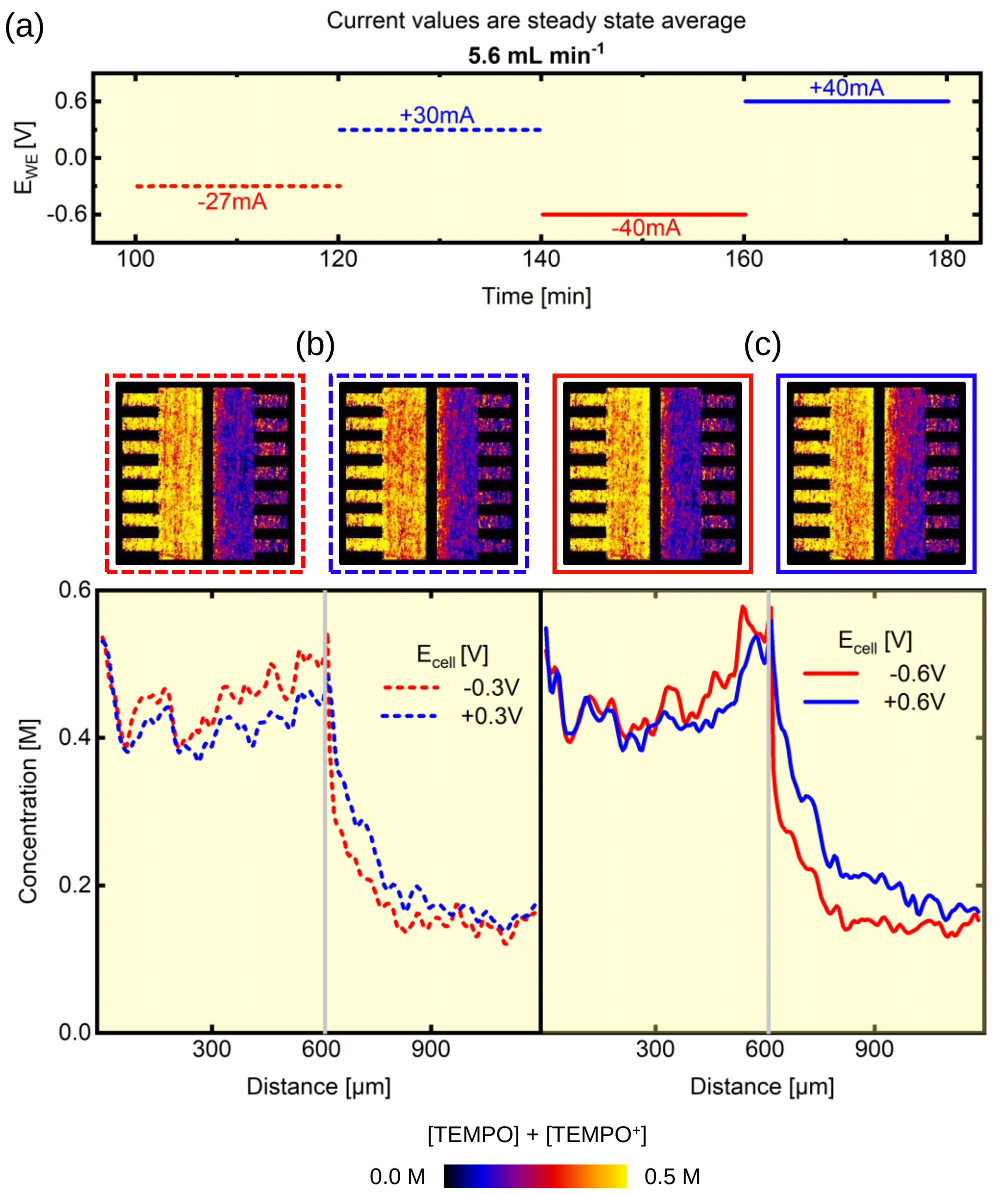

Figure S1: Operando imaging of the active species transport in the NEUTRA beamline with the low attenuating KPF_6 supporting salt at a lower flow rate. (a) Electrochemical sequence over time showing the applied potential steps and measured averaged current output at an inlet flow rate of 5.6 mL min^{-1}. (b-c) Cumulative active species ($TEMPO/TEMPO^+$) concentration profiles over the electrode thickness at an inlet flow rate of 5.6 mL min^{-1}. The averaged snapshots of the cell after image processing and the concentration profiles are shown for various applied potential steps: (b) -0.3 V and +0.3 V, and (c) -0.6 V and +0.6 V.

Supplementary Note 2 – Detailed electrochemical plots

NEUTRA - PF_6^- experiment

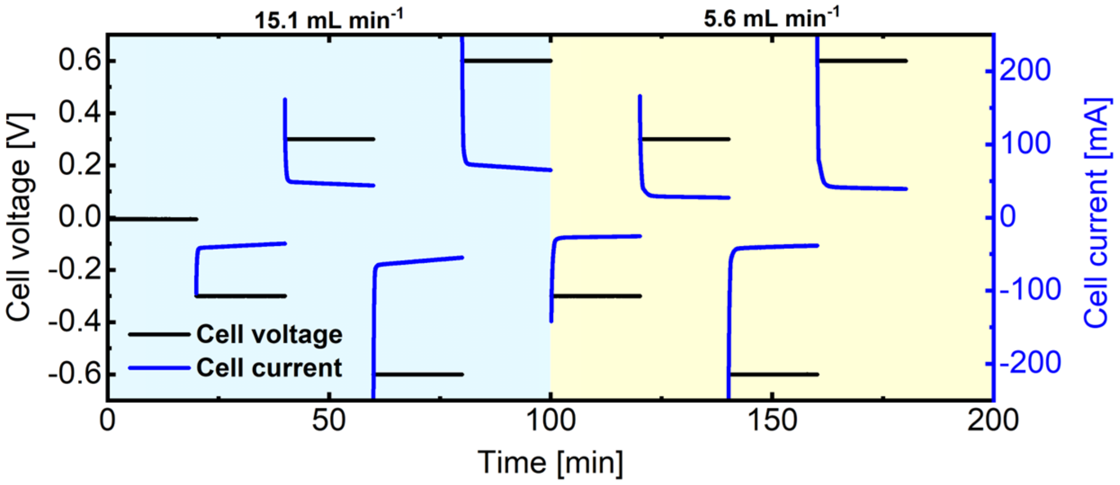

Figure S2: Electrochemical sequence over time showing the applied potential steps and measured current output at two inlet flow rates of 15.1 mL min^{-1} and 5.6 mL min^{-1} with the low attenuating KPF_6 supporting salt in the NEUTRA beamline.

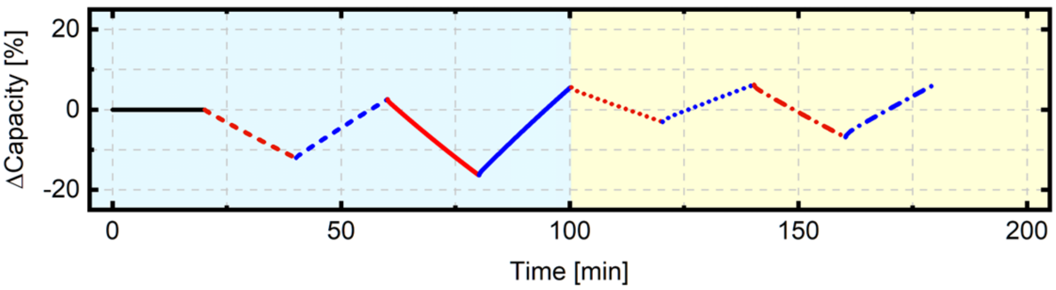

Figure S3: Capacity change over time for various applied potential steps and two inlet flow rates of 15.1 mL min^{-1} and 5.6 mL min^{-1} with the low attenuating KPF_6 supporting salt in the NEUTRA beamline.

NEUTRA - BF_4^- experiment

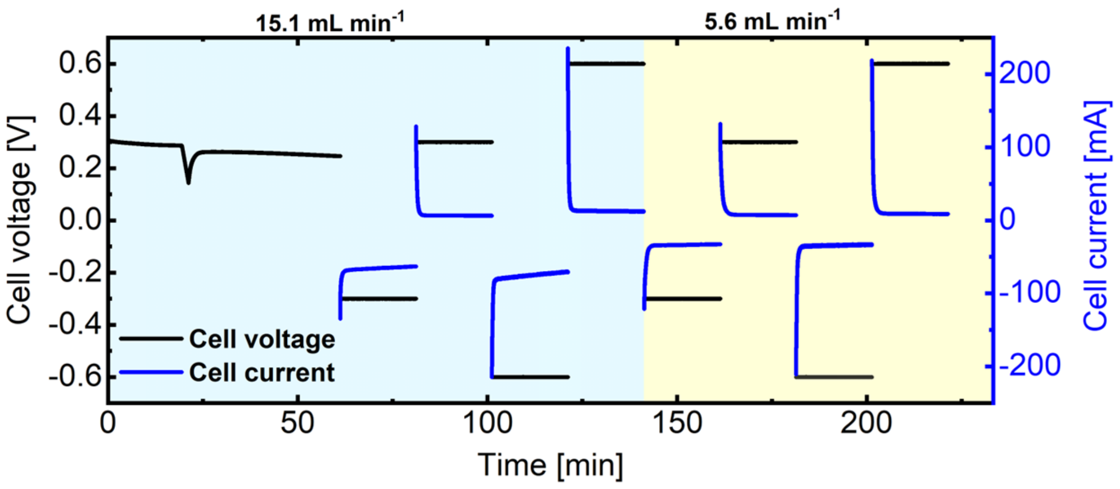

Figure S4: Electrochemical sequence over time showing the applied potential steps and measured current output at two inlet flow rates of 15.1 mL min^{-1} and 5.6 mL min^{-1} with the neutron attenuating BF_4^- supporting ion in the NEUTRA beamline.

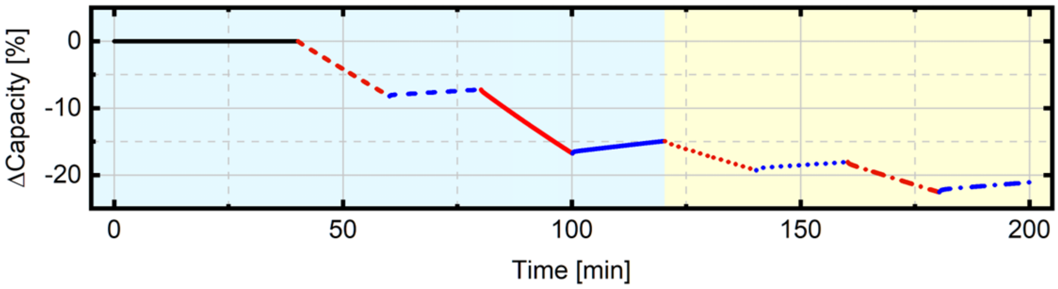

Figure S5: Capacity change over time for various applied potential steps and two inlet flow rates of 15.1 mL min^{-1} and 5.6 mL min^{-1} with the neutron attenuating BF_4^- supporting ion in the NEUTRA beamline.

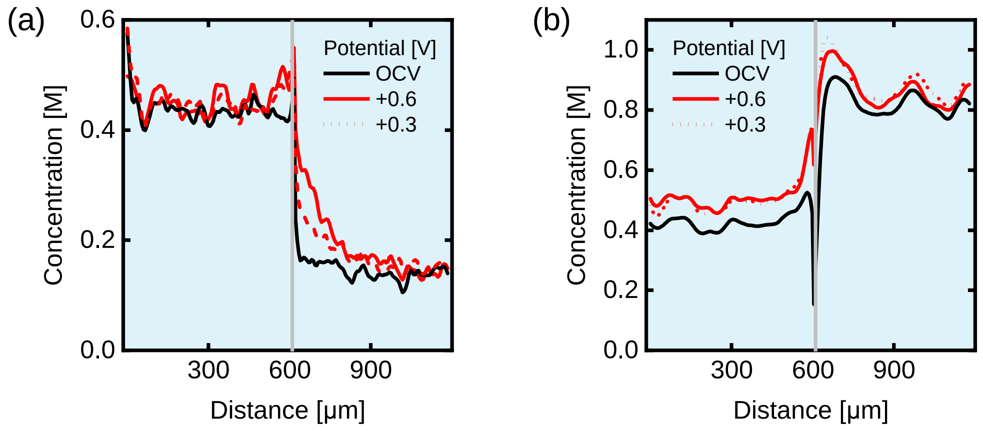

Figure S6: (a) Cumulative active species (TEMPO/$TEMPO^+$) concentration profiles over the electrode thickness at an inlet flow rate of 15.1 mL min^{-1} at applied potential steps of OCV, +0.3 V and +0.6 V. (b) Cumulative active species (TEMPO/$TEMPO^+$) and BF_4^- supporting ion concentration profiles over the electrode thickness at an inlet flow rate of 15.1 mL min^{-1} at applied potential steps of OCV, +0.3 V and +0.6 V.

ICON - BF_4^- experiment

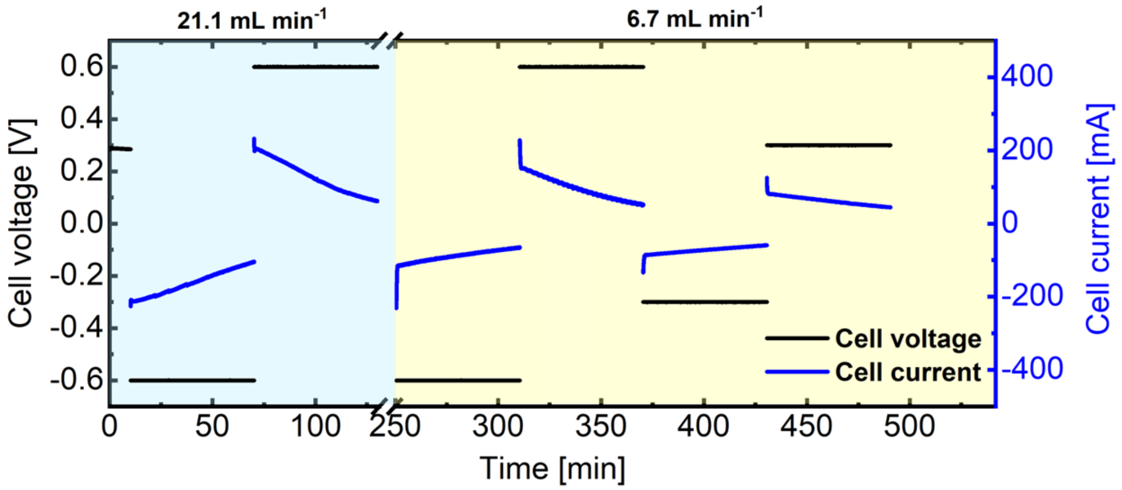

Figure S7: Electrochemical sequence over time showing the applied potential steps and measured current output at two inlet flow rates of 21.1 mL min^{-1} and 6.7 mL min^{-1} with the neutron attenuating BF_4^- supporting ion in the ICON beamline.

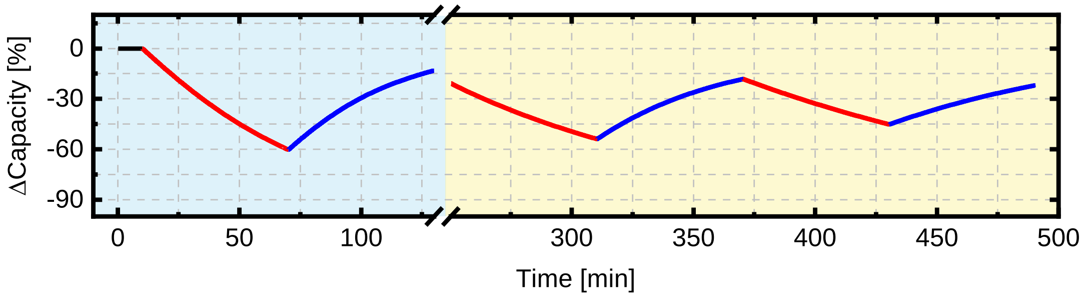

Figure S8: Capacity change over time for various applied potential steps and two inlet flow rates of 21.1 mL min^{-1} and 6.7 mL min^{-1} with the neutron attenuating BF_4^- supporting ion in the ICON beamline.

Supplementary Note 3 – Experimental design considerations

Reactor materials and housing

When designing an experiment utilizing neutron radiography, the design of the electrochemical reactor needs to be considered. The importance of the material for the reactor lies in the fraction of the neutron beam it will attenuate, the higher the attenuation of the neutron beam, the lower the intensity of the collected neutron, and the lower the image quality and imaging sensitivity. In our experimental setup, the neutron beam went through gaskets made from polytetrafluorethylene and through flow fields composed of 3.18 mm thick resin-impregnated graphite plates, both commonly used in redox flow batteries due to their physical and chemical properties. Where most of the elements in the periodic table have a low neutron attenuation, few elements have a high attenuation with neutrons such as hydrogen, lithium, boron, cadmium and gadolinium[1]. From a chemical point of view, the use of polytetrafluorethylene as gasket materials reduces the beam attenuation compared to standard hydrocarbon polymers by replacing the highly attenuating hydrogen centres by fluorine atoms. On the other hand, flow fields are usually manufactured by compressing graphite and injecting phenolic resin in the remaining void space (~20% volume) [2]. The hydrogen atoms present in the phenolic resin however do attenuate the neutron beam. To reduce the attenuation of the beam from reactor materials and housing and to increase the sensitivity of the technique towards the area of interest, the amount of reactor component material (flow field and gasket) in the direction of the neutron beam before the sample was reduced (Figure 1c-d). To sum-up, the material selection for the casing materials should be carefully considered and the physicochemical requirements for the electrochemical application should overlap with the needs for proper neutron imaging. The attenuation of the neutron beam for common reactor components is provided in Table S1.

Table S1: Neutron attenuation coefficients of for commonly used reactor components to guide reactor design for neutron radiography experiments. The neutron attenuation coefficients were calculated for both the NEUTRA and ICON beamlines.

Reactor material	Chemical formula	Neutron attenuation coefficient NEUTRA [cm^{-1}]	Neutron attenuation coefficient ICON [cm^{-1}]
Polytetrafluorethylene gaskets	$(C_2F_4)_x$	0.300	0.318
Polypropylene body	$(C_3H_6)_x$	4.129	5.225
Graphite resin flow field (20% phenolic resin/ 80% graphite)	20% $(C_6H_6O{\cdot}CH_2O)_x$ 80% C	1.058	1.270
Stainless steel	73% Fe, 18% Cr, 8% Ni, 1% Si	1.049	1.203
Aluminum	99% Al, 1% Mn	0.104	0.118

Electrolyte composition

The composition of the salt and active materials, as well as their combination, is important to ensure high selectivity with neutron radiography. In this work, we studied non-aqueous RFBs that typically use fluorinated anions such as hexafluorophosphate (PF_6^-) or tetrafluoroborate (BF_4^-). KPF_6 is composed of elements having high neutron transmissions and therefore shows negligible neutron attenuation compared to the redox active molecules employed in this study (TEMPO/$TEMPO^+$). Hence, the low attenuation of KPF_6 enables the selective imaging of redox active molecules, even in systems with complex reactive mass transport phenomena. On the contrary, BF_4^- contains boron, which strongly

attenuates the neutron beam. Therefore, when utilizing BF_4^- as the supporting salt under white beam conditions, selective imaging of TEMPO/TEMPO$^+$ becomes challenging, resulting in the neutron image representing the sum of BF_4^- + TEMPO + TEMPO$^+$ concentrations. Accordingly, to guide the redox flow battery community in the right combinations of redox system and supporting salt, we listed the neutron attenuation with commonly used supporting salts and redox active molecules in **Table S2**.

Table S2: Neutron attenuation for coefficients of commonly used supporting salts and redox active molecules used in redox flow batteries to show their potential for neutron radiography. The effective neutron attenuation coefficients were calculated for both the NEUTRA and ICON beamlines using a concentration of 1 M in deuterated acetonitrile (corrected for the attenuation of deuterated acetonitrile assuming the molecules are fully dissolved, and the volume of the solution does not change).

Common redox molecules	Chemical formula	Effective neutron attenuation coefficient NEUTRA [cm^{-1}]	Effective neutron attenuation coefficient ICON [cm^{-1}]
Anthraquinone	$C_{14}H_8O_2$	0.292	0.362
Phenazine	$C_{12}H_8N_2$	0.295	0.368
Cyclopropenium	$C_9H_{18}N_3$	0.600	0.757
Methyl phenothiazine	$C_{13}H_{11}NS$	0.384	0.481
Benzothiadiazoles	$C_6H_4N_2S$	0.1558	0.193
Methyl viologen	$C_{12}H_{14}N_2$	0.479	0.602
Ferrocene	$C_{10}H_{10}Fe$	0.344	0.432
Ferricyanide	$[Fe(CN)_6]^{3-}$	0.069	0.078
Chromium	Cr, CrO_4^-, $HCrO_4^-$	0.004, 0.013, 0.044	0.005, 0.015, 0.054
Iron	Fe	0.008	0.009
Hydrogen	H^+, H_2	0.031, 0.061	0.039, 0.078
Vanadium	V, VO^{2+}, VO_2^+	0.006, 0.008, 0.010	0.008, 0.010, 0.013
Zinc	Zn^{2+}	0.003	0.004
Bromide	Br^-_x	0.006	0.009
Iodide	I_x^-	0.006	0.008
Polysulfide	S_x^{2-}	0.001	0.001
Heteropolyacid	$P_2W_{18}O_{62}^{6-}$	0.386	0.516
Riboflavin phosphate	$C_{17}H_{20}N_4O_9P^-$	0.716	0.894
**Common supporting salts**	**Chemical formula**	**Effective neutron attenuation coefficient NEUTRA [cm^{-1}]**	**Effective neutron attenuation coefficient ICON [cm^{-1}]**
KPF_6	KPF_6	0.019	0.020
$TBAPF_6$	$C_{16}H_{36}NPF_6$	1.174	1.483
$NaBF_4$	$NaBF_4$	0.429	0.714
$TBABF_4$	$C_{16}H_{36}NBF_4$	1.585	2.178
LiTFSI	$LiC_2NO_4F_6S_2$	0.077	0.106
$LiPF_6$	$LiPF_6$	0.055	0.082
$LiBF_4$	$LiBF_4$	0.466	0.776
Li^+	Li^+	0.039	0.065
TBA^+	$C_{16}H_{36}N^+$	1.158	1.466
$TFSI^-$	$C_2NO_4F_6S_2^-$	0.038	0.041
K^+	K^+	0.002	0.003
Na^+	Na^+	0.002	0.003
BF_4^-	BF_4^-	0.427	0.712
PF_6^-	PF_6^-	0.016	0.017
ClO_4^-	ClO_4^-	0.037	0.050
Cl^-	Cl^-	0.028	0.040
SO_4^{2-}	SO_4^{2-}	0.100	0.106
Hydroxide	OH^-	0.033	0.042

Moreover, not only the combination and composition of the salt and active materials is important, but also which specific imaging configuration is utilized. In our study, we employed two types of neutron imaging configurations: (1) white beam with thermal neutrons (NEUTRA beamline), and (2) cold neutrons coupled with the time-of-flight technique (ICON beamline). ICON offers neutron energy selective imaging by leveraging the energy-dependency of neutron attenuation in materials. This means that if the supporting salt and redox molecules have varying attenuations at different neutron energies, their concentrations can be distinguished, allowing for high imaging selectivity. On the other hand, NEUTRA relies on white beam imaging, resulting in a neutron image that reflects the total neutron attenuation of all mixed components in the beam path. Consequently, the electrolyte composition should depend on the imaging configuration of interest.

Supplementary Note 4 – Image processing

NEUTRA

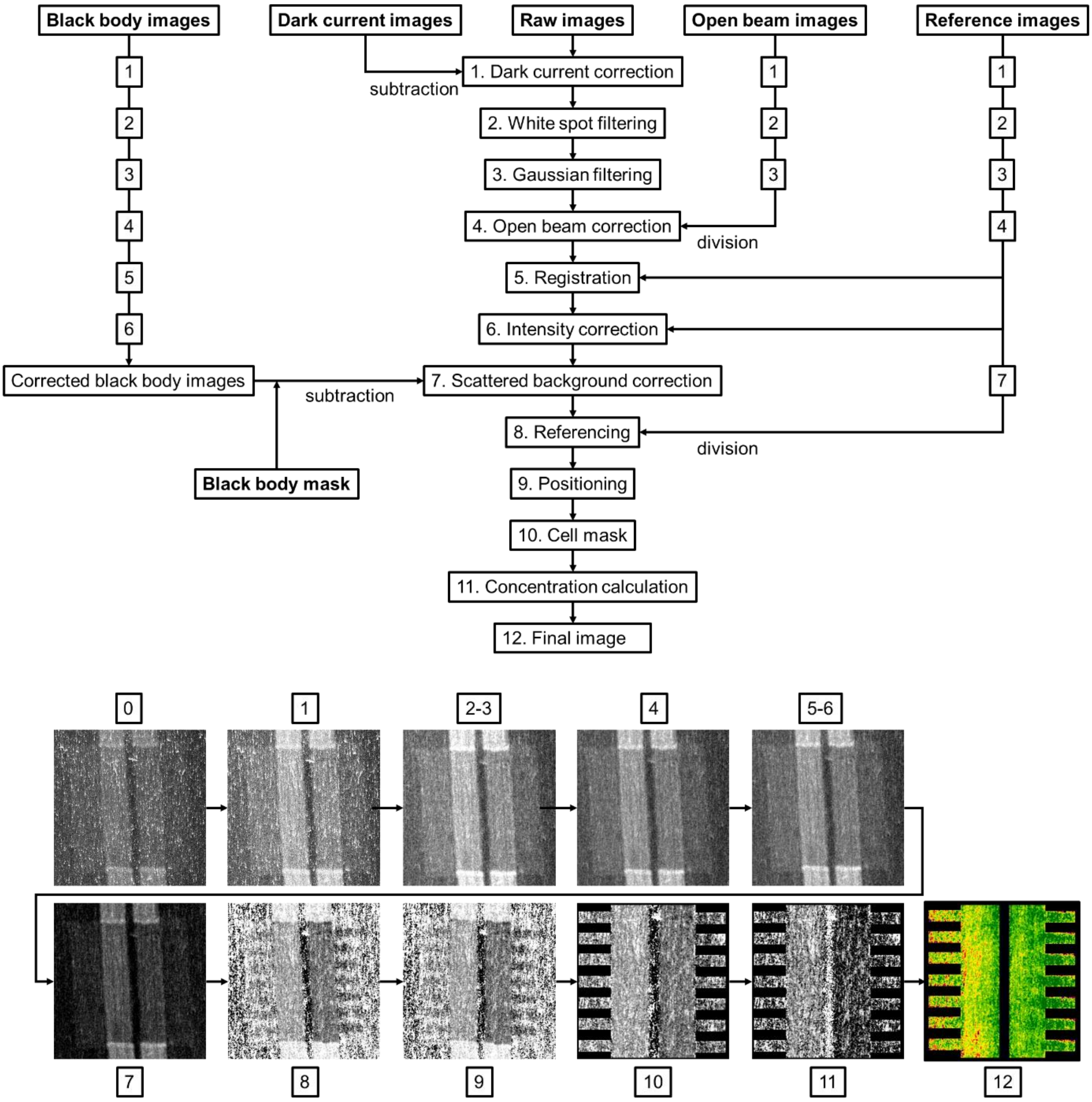

Figure S9: Image processing sequence for the experiments conducted at the NEUTRA beamline and the resulting images after each processing step.

References

1. Peng, Z. & Tiejun, Z. Neutron Radiography. in *Methods of Measuring Moisture in Building Materials and Structures* (ed. Nilsson, L.-O.) vol. 26 141–155 (Springer International Publishing, Cham, 2018).
2. GAB Neumann GmbH. Impervious Graphite. *Heat Exchangers and Components in Graphite and Silicon CarbideGAB Neumann GmbH* https://www.gab-neumann.com/impervious-graphite (2017).
