# Supplementary material for: Quantifying concentration distributions in redox flow batteries with neutron radiography
Source: Nat Commun. 2024 Sep 5;15:7434. doi: 10.1038/s41467-024-50120-7 (PMC11377732; doi:10.1038/s41467-024-50120-7)
Supplement: Supplementary file 3 — Description of Additional Supplementary Files [file 41467_2024_50120_MOESM3_ESM.docx]

**File Name:** Supplementary Video 1

**Description:** Neutron imaging experiments at the NEUTRA beamline using 0.2 M KPF_6_ supporting salt with 0.5 M TEMPO/TEMPO^+^PF_6_^-^ solution at 50% SoC in the counter electrode side (left) and 0.2 M TEMPO/TEMPO^+^PF_6_^-^ solution at 50% SoC in the working electrode side (right). The radiographs (left side of the video) are given with an active-species concentration color scale and the flow rate, while voltage-time and current-time plots (right side of the video) are synchronized to the radiographs.

**File Name:** Supplementary Video 2

**Description:** Neutron imaging experiments at the NEUTRA beamline using BF_4_^-^ counter-ion with 0.5 M TEMPO solution in the counter electrode side (left) and 0.5 M TEMPO/TEMPO^+^BF_4_^-^ solution in the working electrode side (right). The radiographs (left side of the video) are given with a cumulative (active species + counter-ion) concentration color scale and the flow rate, while voltage-time and current-time plots (right side of the video) are synchronized to the radiographs.
